# Heterothallism and potential hybridization events inferred for twenty-two yellow morel species

**DOI:** 10.1186/s43008-020-0027-1

**Published:** 2020-02-12

**Authors:** Xi-Hui Du, Dongmei Wu, Heng Kang, Hanchen Wang, Nan Xu, Tingting Li, Keliang Chen

**Affiliations:** 1 College of Life Sciences, Chongqing Normal University, Chongqing, 401331 China; 2 Biotechnology Research Institute, Xinjiang Academy Agricultural Reclamation of Sciences, Shihezi, 832000 China; 3 Institute of Applied Mycology, Huazhong Agricultural University, Wuhan, 430070 Hubei China

**Keywords:** *Morchella*, Esculenta clade, Mating type, Conflict, Phylogeny, Reproductive mode, IGS, *F1*, Gene transfer

## Abstract

Mating-type genes are central to sexual reproduction in ascomycete fungi and result in the establishment of reproductive barriers. Together with hybridization, they both play important roles in the evolution of fungi. Recently, potential hybridization events and MAT genes were separately found in the Elata Clade of *Morchella*. Herein, we characterized the *MAT1–1-1* and *MAT1–2-1* genes of twenty-two species in the Esculenta Clade, another main group in the genus *Morchella,* and proved heterothallism to be the predominant mating strategy among the twenty-two species tested. Ascospores of these species were multi-nuclear and had many mitochondrial nucleoids. The number of ascospore nuclei might be positively related with the species distribution range. Phylogenetic analyses of *MAT1–1-1*, *MAT1–2-1,* intergenic spacer (IGS), and partial histone acetyltransferase ELP3 (*F1*) were performed and compared with the species phylogeny framework derived from the ribosomal internal transcribed spacer region (ITS) and translation elongation factor 1-alpha (*EF1-a*) to evaluate their species delimitation ability and investigate potential hybridization events. Conflicting topologies among these genes genealogies and the species phylogeny were revealed and hybridization events were detected between several species. Different evolutionary patterns were suggested for MAT genes between the Esculenta and the Elata Clades. Complex evolutionary trajectories of *MAT1–1-1*, *MAT1–2-1*, *F1* and IGS in the Esculenta Clade were highlighted. These findings contribute to a better understanding of the importance of hybridization and gene transfer in *Morchella* and especially for the appearance of reproductive modes during its evolutionary process.

## INTRODUCTION

In fungi, sexual reproduction is regulated by genomic regions called mating-type (MAT) loci (Kronstad and Staben 1997; Debuchy et al. 2010; Casselton and Feldbrügge 2010), which operate on the establishment of reproductive barriers, determine mating compatibility and result in speciation (Coppin et al. 1997; Lee et al. 2010; Palumbi 2009; Swanson and Vacquier 2002). In filamentous ascomycetes, the mating-type system is bipolar and has a single MAT locus called MAT1 with two alternate forms as master regulators of sexual reproduction: the *MAT1–1-1* and the *MAT1–2-1* genes, respectively, encoding for alpha-box and a high mobility group (HMG) domain proteins (Metzenberg and Glass 1990; Debuchy et al. 2010). Due to their dissimilar sequences, the two forms of the MAT locus are referred to as idiomorphs instead of alleles (Metzenberg and Glass 1990). Filamentous ascomycetes have two main sexual reproductive modes: homothallism and heterothallism. Homothallic fungi are self-fertile and can complete the sexual cycle without a mating partner. Typically, a single homothallic strain harbors both MAT idiomorphs (linked or unlinked) in the same haploid nucleus (Debuchy et al. 2010). On the contrary, heterothallic fungi are self-sterile, contain only one of the two MAT genes, and need the participation of an opposite mating type partner to reproduce.

Reproductive modes of fungi are assumed to have a great influence on the evolutionary trajectory of their genomes (Burt 2000). In recent years, MAT genes have gained significant attention in evolutionary biology: on the one hand because of the general relationship between the evolution of reproductive genes and the reproductive modes of individuals (Paoletti et al. 2006; Walters and Harrison 2008; Wik et al. 2008), and on the other hand because MAT genes are known to be evolutionarily dynamic with high evolutionary rates (Gioti et al. 2012; Martin et al. 2013; Sun and Heitman 2015). Therefore, MAT gene sequences have often been used to study evolutionary trends of mating systems (Fraser et al. 2007; Wik et al. 2008) and population genetics (Zhan et al. 2007; Groenewald et al. 2007). Because of the high evolutionary rates of MAT genes, some studies inferred a phylogenetic relationship suggesting that the phylogenies of MAT genes were consistent with other genes (Groenewald et al. 2006; Yokoyama et al. 2006; Lopes et al. 2017; O’Donnell et al. 2004; Duong et al. 2013; Du et al. 2017) whereas others found conflicting topologies (Wik et al. 2008; Strandberg et al. 2010).

True morels belong to the genus *Morchella* (*Ascomycota*, *Pezizales*, *Morchellaceae*), are widely known for their iconic edible ascomata and are among the world’s most prized edible fungi. The *Morchella* genus comprises the Esculenta Clade (yellow morels), the Elata Clade (black morels), and the Rufobrunnea Clade (blushing morels) with more than seventy species in total (Du et al. 2019b). Considering their important economic value, understanding the reproductive biology of these species is not only of fundamental but also of applied relevance, for example for artificial cultivation and cultivar breeding. However, knowledge about MAT genes in *Morchella* is currently limited to fourteen species from the Elata Clade. For these species, a heterothallic sexual cycle has been proposed while the phylogeny of MAT genes is consistent with other genes (Du et al. 2017). Species in the Esculenta Clade not only have a distinguished morphology from the Elata Clade, but also favor different ecological niches (Du et al. 2012; Kuo et al. 2012). Their reproductive modes are still unknown and the usefulness of MAT genes for species identification of MAT genes needs to be investigated. Additionally, considering that multiple hybridization events were detected in the Elata Clade (Du et al. 2016, 2019a), the *F1* and IGS genes were chosen to assess whether they can be used for species identification and for the identification of potential hybridization events in the Esculenta Clade, based on previous studies focusing on the Elata Clade (Du et al. 2016) and the genus *Fusarium* (Mbofung et al. 2006).

The present study therefore sought: (i) to isolate and characterize the *MAT1–1-1* and *MAT1–2-1* genes of twenty-two yellow morel species; (ii) to illuminate the reproductive mode of these twenty-two species; (iii) to evaluate the *MAT1–1-1* and *MAT1–2-1* polymorphism intra- and interspecies; (iv) to infer the usefulness of the *MAT1–1-1* and *MAT1–2-1* genes as phylogenetic markers in the Esculenta Clade; and (v) to investigate the potential hybridization events among these species. Considering the increased interest in morel cultivation in China (Du et al. 2019a), description of the reproductive modes of these species is of high applied value.

## MATERIAL AND METHODS

### Obtaining of *MAT1–1-1* and *MAT1–2-1* sequences

The genome of *Mes-*21 was sequenced using an Illumina HiSeq 2500 Genome Analyzer (Illumina Inc., USA) by Genoseq Biotechnology Co., Ltd. (Wuhan, China) based on highly purified total genomic DNA isolated from the fungal mycelia culture. The genome was assembled by SOAPdeNovo2 (Zerbino et al. 2008) to first construct contigs based on the short insert libraries, then joining these to scaffolds using paired-end information, followed by local reassembly of unresolved gap regions. In order to detect the best assembly(s), kmer levels were respectively run at 41, 43, 45, 49 and 55, and an optimised k-mer size of 41 was suggested. The genome data of Mes-*21* generated in this project has been deposited at DDBJ/EMBL/GenBank under the submission no. SUB6606995.

*MAT1–1-1* and *MAT1–2-1* were identified using BLASTn and BLASTx against the NCBI nucleotide and protein database by sequence similarity searches (http://www.ncbi.nlm.nih.gov/BLAST/) in the genome of *Mes-*21. The alpha-box- and HMG-containing sequences of *M. importuna* (KY508074 and KY508167) and *M. sextelata* (KY508145 and KY508228) from the Elata Clade were chosen for BLAST analysis of *MAT1–1-1* and *MAT1–2-1* in the *Mes*-21 genome.

### Fungal materials and species identification

Eighty-three samples collected in China, Croatia and France between 2016 and 2019 were used in this study. Based on two-gene phylogenetic species recognition analysis via nuc-rDNA internal transcribed spacer region (ITS) and translation elongation factor 1-α (*EF1-a*) gene, the samples were characterized and belonged to twenty-two phylogenetic species of Esculenta Clade in the genus *Morchella*, namely *M. steppicola*, *M. yangii*, *M. yishuica*, *M. clivicola*, *M. dunensis*, *M. palazonii*, *M. americana*, *M. esculenta*, *M. galilaea*, *Mes*-6, *Mes*-9, *Mes*-10, *Mes*-15, *Mes*-19, *Mes*-20, *Mes*-21, *Mes*-22, *Mes*-23, *Mes*-24, *Mes*-25, *Mes*-26, and *Mes*-27 (Table 1). At least two samples from each species were selected, except for *M. steppicola* and *Mes*-24 since only one sample was obtained of each. All the collections used in the present study were dried with silica and housed in the Fungal Herbarium of Chongqing Normal University, Chongqing City, China (FCNU). Information on these samples is detailed in Table 1.

**Table 1:** Fungal names, specimen voucher, locations and GenBank accession numbers

Taxon	Specimen voucher	Location	GenBank accession number						Pi of MAT1–1-1/ Pi of MAT1–2-1 (within each species)
			ITS	*EF1-a*	*MAT1–1-1*	*MAT1–2-1*	*F1*	IGS	
*M. amerciana*	FCNU1033	France	MN513710	MN513637	MN513853	MN513936	MN513445	×^a^	0.0000/0.0012
*M. amerciana*	FCNU1109	France	MN513706	MN513633	MN513849	MN513932	MN513442	MN513774	
*M. amerciana*	FCNU1110	Croatia	MN513664	MN513737	MN513883	MN513966	MN513470	×	
*M. amerciana*	FCNU1111	France	MN513708	MN513635	MN513851	MN513934	×	×	
*M. amerciana*	FCNU1112	France	MN513709	MN513636	MN513852	MN513935	MN513444	×	
*M. clivicola*	FCNU1019	Sichuan, China	MK321870	MK321918	MN513848	MN513931	MN513441	MN513773	0.0031/0.0012
*M. clivicola*	FCNU1021	Hubei, China	MK321871	MK321919	MN513861	MN513944	MN513451	MN513782	
M. *dunensis*	FCNU1029	Xinjiang, China	MK321872	MK321920	MN513907	MN513990	MN513488	MN513824	0.0010/0.0006
*M. dunensis*	FCNU1030	Xinjiang, China	MK321873	MK321921	MN513906	MN513989	MN513487	MN513823	
*M. dunensis*	FCNU1067	France	MN513757	MN513684	MN513904	MN513987	MN513486	MN513821	
*M. dunensis*	FCNU1068	France	MN513758	MN513685	MN513905	MN513988	×	MN513822	
*M. esculenta*	FCNU1038	Henan, China	MN513733	MN513660	MN513877	MN513960	MN513465	MN513796	0.0018/0.0005
*M. esculenta*	FCNU1039	Henan, China	MN513735	MN513662	MN513881	MN513964	MN513469	MN513800	
*M. esculenta*	FCNU1040	Liaoning, China	MN513719	MN513646	MN513863	MN513946	MN513453	×	
*M. esculenta*	FCNU1041	France	MN513747	MN513674	MN513893	MN513976	MN513477	MN513810	
*M. esculenta*	FCNU1042	Shaanxi, China	MN513696	MN513623	MN513838	MN513921	MN513431	×	
*M. galilaea*	FCNU1061	Kenya	MN513736	MN513663	MN513882	MN513965	×	MN513801	0.0000/0.0000
*M. galilaea*	FCNU1062	Sichuan, China	MN513705	MN513632	MN513847	MN513930	MN513440	MN513772	
*M. galilaea*	FCNU1063	Yunnan, China	MN513699	MN513626	MN513841	MN513924	MN513434	MN513766	
*M. galilaea*	FCNU1064	Yunnan, China	MN513700	MN513627	MN513842	MN513925	MN513435	MN513767	
*M. palazonii*	FCNU1031	Xinjiang, China	MN513700	MN513627	MN513908	MN513991	MN513489	MN513825	0.0107/0.0000
*M. palazonii*	FCNU1103	Xinjiang, China	MN513759	MN513686	MN513909	MN513992	×	MN513826	
M. *steppicola*	FCNU1107	Croatia	MN513738	MN513665	MN513884	MN513967	×	×	N
*M. yangii*	FCNU1012	Henan, China	MK321878	MK321926	MN513880	MN513963	MN513468	MN513799	0.0011/0.0000
*M. yangii*	FCNU1013	Henan, China	MK321885	MK321933	MN513912	MN513995	MN513492	MN513829	
*M. yangii*	FCNU1014	Henan, China	MK321877	MK321925	MN513878	MN513961	MN513466	MN513797	
*M. yangii*	FCNU1050	Henan, China	MN513713	MN513640	MN513856	MN513939	MN513447	MN513777	
*M. yangii*	FCNU1051	Henan, China	MN513714	MN513641	MN513857	MN513940	MN513448	MN513778	
*M. yangii*	FCNU1052	Shaanxi, China	MN513727	MN513654	MN513871	MN513954	MN513460	MN513790	
*M. yangii*	FCNU1053	Henan, China	MN513731	MN513658	MN513875	MN513958	MN513464	MN513794	
*M. yangii*	FCNU1054	Henan, China	MN513704	MN513631	MN513846	MN513929	MN513439	MN513771	
*M. yangii*	FCNU1055	Henan, China	MN513712	MN513639	MN513855	MN513938	MN513446	MN513776	
*M. yishuica*	FCNU1017	Shandong, China	MK321882	MK321930	MN513897	MN513980	MN513480	MN513814	0.0000/0.0000
*M. yishuica*	FCNU1105	Shandong, China	MN513751	MN513678	MN513898	MN513981	MN513481	MN513815	
*M. yishuica*	FCNU1106	Shandong, China	MN513750	MN513677	MN513896	MN513979	×	MN513813	
*Mes* − 6	FCNU1034	Shanxi, China	MN513693	MN513620	MN513835	MN513918	MN513428	×	0.0000/0.0014
*Mes* − 6	FCNU1035	Hebei, China	MN513695	MN513622	MN513837	MN513920	MN513430	×	
*Mes* − 6	FCNU1036	Qinghai, China	MN513697	MN513624	MN513839	MN513922	MN513432	MN513764	
*Mes* − 6	FCNU1037	Qinghai, China	MN513698	MN513625	MN513840	MN513923	MN513433	MN513765	
*Mes* − 9	FCNU1043	Shandong, China	MN513744	MN513671	MN513890	MN513973	×	MN513807	0.0015/0.0010
*Mes* − 9	FCNU1044	Shandong, China	MN513745	MN513672	MN513891	MN513974	MN513475	MN513808	
*Mes* − 9	FCNU1045	Shandong, China	MN513748	MN513675	MN513894	MN513977	MN513478	MN513811	
*Mes* − 9	FCNU1046	Shandong, China	MN513749	MN513676	MN513895	MN513978	MN513479	MN513812	
*Mes* − 10	FCNU1048	Yunnan, China	MN513692	MN513619	MN513833	MN513916	MN513427	×	0.0031/0.0000
*Mes* − 10	FCNU1049	Henan, China	MN513711	MN513638	MN513854	MN513937	×	MN513775	
*Mes* − 15	FCNU1056	Yunnan, China	MN513752	MN513679	MN513899	MN513982	MN513482	MN513816	0.0000/0.0005
*Mes* − 15	FCNU1057	Yunnan, China	MN513752	MN513683	MN513899	MN513982	MN513482	MN513816	
*Mes* − 15	FCNU1058	Sichuan, China	MN513739	MN513666	MN513885	MN513968	MN513471	MN513802	
*Mes* − 15	FCNU1059	Yunnan, China	MN513694	MN513621	MN513836	MN513919	MN513429	×	
*Mes* − 15	FCNU1060	Guizhou, China	MN513718	MN513645	MN513862	MN513945	MN513452	MN513783	
*Mes* − 19	FCNU1069	Hubei, China	MN513716	MN513643	MN513859	MN513942	MN513450	MN513780	0.0008/0.0004
*Mes* − 19	FCNU1070	Henan, China	MN513760	MN513687	MN513910	MN513993	MN513490	MN513827	
*Mes* − 19	FCNU1071	Chongqing, China	MN513726	MN513653	MN513870	MN513953	MN513459	MN513789	
*Mes* − 19	FCNU1072	Chongqing, China	MN513725	MN513652	MN513869	MN513952	MN513458	MN513788	
*Mes* − 19	FCNU1073	Henan, China	MN513724	MN513651	MN513868	MN513951	MN513457	MN513787	
*Mes* − 19	FCNU1074	Yunnan, China	MN513753	MN513680	MN513900	MN513983	MN513483	MN513817	
*Mes* − 20	FCNU1075	Henan, China	MN513761	MN513688	MN513911	MN513994	MN513491	MN513828	0.0006/0.0010
*Mes* − 20	FCNU1076	Henan, China	MN513729	MN513656	MN513873	MN513956	MN513462	MN513792	
*Mes* − 20	FCNU1077	Henan, China	MN513762	MN513689	MN513913	MN513996	MN513493	MN513830	
*Mes* − 20	FCNU1078	Henan, China	MN513763	MN513690	MN513914	MN513997	MN513494	MN513831	
*Mes* − 20	FCNU1079	Shandong, China	MN513746	MN513673	MN513892	MN513975	MN513476	MN513809	
*Mes* − 21	FCNU1080	Henan, China	MN513732	MN513659	MN513876	MN513959	×	MN513795	0.0006/0.0007
*Mes* − 21	FCNU1081	Henan, China	MN513734	MN513661	MN513879	MN513962	MN513467	MN513798	
*Mes* − 21	FCNU1082	Gansu, China	MN513707	MN513634	MN513850	MN513933	MN513443	×	
*Mes* − 21	FCNU1083	Henan, China	MN513703	MN513630	MN513845	MN513928	MN513438	MN513770	
*Mes* − 21	FCNU1084	Henan, China	MN513702	MN513629	MN513844	MN513927	MN513437	MN513769	
*Mes* − 22	FCNU1085	Yunnan, China	MN513754	MN513681	MN513901	MN513984	×	MN513818	0.0000/0.0069
*Mes* − 22	FCNU1086	Yunnan, China	MN513755	MN513682	MN513902	MN513985	MN513484	MN513819	
*Mes* − 22	FCNU1088	Hubei, China	MN513715	MN513642	MN513858	MN513941	MN513449	MN513779	
*Mes* − 22	FCNU1089	Hubei, China	MN513717	MN513644	MN513860	MN513943	×	MN513781	
*Mes* − 23	FCNU1087	Guangdong, China	MN513701	MN513628	MN513843	MN513926	MN513436	MN513768	0.0000/0.0000
*Mes* − 23	FCNU1090	Sichuan, China	MN513740	MN513667	MN513886	MN513969	MN513472	MN513803	
*Mes* − 23	FCNU1091	Sichuan, China	MN513741	MN513668	MN513887	MN513970	MN513473	MN513804	
*Mes* − 23	FCNU1092	Hubei, China	MN513722	MN513649	MN513866	MN513949	×	MN513785	
*Mes* − 23	FCNU1093	Zhejiang, China	MN513728	MN513655	MN513872	MN513955	MN513461	MN513791	
*Mes* − 23	FCNU1094	Hubei, China	MN513723	MN513650	MN513867	MN513950	MN513456	MN513786	
*Mes* − 24	HMAS96865	Beijing, China	JQ322043	JQ322002	MN513834	MN513917	×	×	N
*Mes* − 25	FCNU1096	Henan, China	MN513730	MN513657	MN513874	MN513957	MN513463	MN513793	0.0000/0.0025
*Mes* − 25	FCNU1097	Sichuan, China	MN513691	MN513618	MN513832	MN513915	MN513426	×	
*Mes* − 26	FCNU1098	Shandong, China	MN513742	MN513669	MN513888	MN513971	MN513474	MN513805	0.0000/0.0000
*Mes* − 26	FCNU1099	Shandong, China	MN513670	MN513743	MN513889	MN513972	×	MN513806	
*Mes* − 27	FCNU1101	Zhejiang, China	MN513720	MN513647	MN513864	MN513947	MN513454	×	0.0000/0.0000
*Mes* − 27	FCNU1108	Zhejiang, China	MN513721	MN513648	MN513865	MN513948	MN513455	MN513784	

^a^: This sample failed to generate amplicons of this gene

### Selection of loci

Two mating-type genes (*MAT1–1-1* and *MAT1–2-1*), herein referred to as reproductive genes, were investigated in this study. The nuc-rDNA internal transcribed spacer region (ITS) and translation elongation factor 1-α (*EF1-a*) gene were used to identify these eight-three samples in this study (Table 1). Additionally, 28S–18S ribosomal intergenic spacer region (IGS) and partial sequences of histone acetyltransferase ELP3 (*F1*) with assumed functions independent of sexual reproduction in *Morchella* were selected for comparative purposes. IGS and *F1* are referred to as non-reproductive genes in this paper.

### Single spore isolates and cultural conditions

Twenty single ascospores were randomly isolated from each species, except for *M. steppicola*, *Mes*-24 and *Mes*-27, because samples from the three species were immature. Ascospores were washed, suspended in sterilized water, and 200 μL of a solution adjusted to a concentration of 200–300 ascospores mL^− 1^ was spread on potato dextrose agar (PDA) (Solarbio, China) and incubated at 23–25 °C for 1–2 days. Single germinated ascospores were picked using a dissecting needle under a dissecting microscope (Zeiss 455,094), and transferred to a new PDA petri dish, and incubated at 23–25 °C for 2 weeks. Then, the mycelium were harvested by scraping the surface of PDA using a clean surgical blade and used for DNA extraction.

### DNA extraction

Samples (mycelium/ascomata) were ground to a fine powder in a 1.5-mL microcentrifuge tube using a Kontes pellet pestle (Kaimu, China). Once pulverized, the samples were suspended in 700 μL CTAB extraction buffer (100 mM Tris–Cl pH 8.4, 1.4 M NaCl, 25 mM EDTA, 2% CTAB), and incubated for 1.5–2.0 h at 65 °C during which time they were gently inverted 3–5 times. After the samples were cooled to room temperature, 700 μL chloroform–isoamyl alcohol (24:1) was added to each tube. The mixture was vortexed briefly, centrifuged at 12,000 *g* for 10 min, and 500 μL of the upper phase was carefully transferred to a new 1.5-mL microcentrifuge tube. After a second chloroform–isoamyl alcohol (24:1) extraction was performed, the supernatant was transferred to a new 1.5-mL microcentrifuge tube and an equal volume of 100% isopropanol at 20 °C was added to each tube. The tube contents were mixed briefly by inversion to obtain a homogeneous solution and then they were stored overnight at 20 °C to precipitate total genomic DNA. After the tubes were warmed to room temperature, they were centrifuged at 12,000 *g* for 10 min and the supernatant was discarded. The DNA pellet was washed consecutively with 70 and 100% ethanol, air-dried and resuspended in 100 μL of sterile double-distilled H_2_O. All genomic DNA samples were stored frozen at − 20 °C.

### PCR amplification and DNA sequencing

The primer sequences of *MAT1–1-1*, *MAT1–2-1*, ITS, *EF1-a*, IGS and *F1* genes are reported in Table 2 and used for the following PCR and DNA sequencing. PCR was carried out on an ABI 2720 Thermal Cycler (Applied Biosystems, Foster City, CA, USA) using the following program: pre-denaturation at 94 °C for 3 min, followed by 35 cycles of denaturation at 94 °C for 30 s, annealing at 53 °C for 30 s and elongation at 72 °C for 30 s. Afterwards, a final elongation at 72 °C for 8 min was performed. PCR products were sent to Kunming Shuoqing Biotech Ltd. to carry out DNA sequencing. Raw sequence data of *MAT1–1-1*, *MAT1–2-1*, ITS, *EF1*-a, IGS and *F1* were edited and aligned with SeqMan implemented in Lasergene v7. 1 (DNASTAR Inc., USA) and automatically aligned with MAFFT v6.853 using the E-INS-i strategy (Katoh et al. 2002). Aligned sequences were visually inspected and manually adjusted using BioEdit v7.0.9. Sequences generated in the present study have been deposited in GenBank under accession numbers MN513618-MN513997 and MN513426-MN513494.

**Table 2:** PCR and sequencing primers used in this study

Locus	Primer	References	Sequence (5′-3′)^a^	Tm
*MAT1–1-1*	EMAT1–1 L	This study	TAGGTAGGTCCCAAGAACACC	50 °C
	EMAT1–1R	This study	GATACCATGGCGAACATTCTG	
*MAT1–2-1*	EMAT1–2 L	This study	CTTGCCACTACGCGGTCTAT	50 °C
	EMAT1–2R	This study	CACGGCTCTGGTATCCATTC	
*EF-1a*	EF-595F	Kauserud and Schumacher (2001)	CGTGACTTCATCAAGAACATG	50 °C
	EF-1R	Du et al. (2012)	GGARGGAAYCATCTTGACGA	
ITS	ITS4	White et al. (1990)	TCCTCCGCTTATTGATATGC	50 °C
	ITS5	White et al. (1990)	GGAAGTAAAAGTCGTAACAAGG	
*F1*	F1F	Du et al. (2016)	GGCTAAGATACGAGCTACGAGA	49 °C
	F1R	Du et al. (2016)	ACATCAATGAGAGCCATTCG	
IGS	IGSYL	This study	CTTACTCCTGCAATCGTAGT	49 °C/50 °C
	IGSYR	This study	TGGTTACCCTGCCTCCAT	

### Identification of species and phylogenetic analysis of all the loci

Considering the difficulty of accurately distinguishing all the morel species according to morphology, species identification of all the samples used in this study was previously performed based on molecular phylogenetic analysis of the ITS rDNA and *EF1*-*a* datasets, which were previously used for species identification in the genus *Morchella* (O’Donnell et al. 2011, Du et al. 2012). For the concatenated and two individual matrices (ITS-*EF1-a*, *MAT1–1-1* and *MAT1–2-1*), the taxon *M. steppicola* was used as the outgroup in this study as inferred by O’Donnell et al. (2011) and Du et al. (2012). Because of our inability to sequence locus *F1* and IGS for *M. steppicola*, *M. americana* was used as the outgroup in the *F1* datasets as inferred by O’Donnell et al. (2011) and Du et al. (2012); whereas mid-rooted trees were generated for the IGS datasets as no suitable taxon could serve as the outgroup. All eighty-three samples from twenty-two species successfully generated amplicons from ITS, *EF1*a, *MAT1–1-1* and *MAT1–2-1*. However, some samples failed to generate amplicons of IGS and *F1* and were shown in Table 1.

Using these generated sequences, phylogenetic analyses were performed on the following datasets: (i) for the combined ITS and *EF1-a* dataset, 83 sequences, 22 species; (ii) for the *MAT1–1-1* dataset, 83 sequences, 22 species; (iii) for the *MAT1–2-1* dataset, 83 sequences, 22 species; (iv) for the IGS dataset, 68 sequences, 20 species; and (v) for the *F1* dataset, 69 sequences, 20 species. The combined nucleotide sequences of ITS and *EF1-a* were used to construct the species tree for all samples in Table 1.

Phylogenetic analyses using a Maximum parsimony (MP) analysis and Maximum Likelihood (ML) analysis were subsequently conducted on PAUP V 4.0b10 (Swofford 2002) and RAxML v7.2.6 (Stamatakis 2006), respectively. For the concatenated two-gene matrices, single-gene analyses were conducted to assess incongruence among individual genes using the MP method (results not shown). Because no well-supported (BS > 70%) conflict was detected among the topologies of the two genes, their sequences were then concatenated together for further analyses. For the two-gene datasets, a partitioned model, with the model (GTR + I + G) for both of genes, was used by defining the sequences of ITS and *EF1-a* as two partitions. For the MP analysis, heuristic searches were conducted with tree bisection–reconnection branch swapping algorithm (TBR) after exclusion of uninformative characters, random sequence additions, and with Multrees option on. Bootstrap values (BP) of the most parsimonious trees were obtained from 1000 replications. Gaps were defined as a fifth character in all analyses. For the ML analyses, all of the parameters were kept at their default settings, except that the model was set as GTRGAMMAI (Stamatakis 2006) which was applied for the concatenated and four individual matrices (ITS-*EF1-a*, *MAT1–1-1*, *MAT1–2-1*, *F1* and IGS), while statistical support was obtained using nonparametric bootstrapping with 1000 replicates. Trees generated by the two analyses were viewed and exported in FigTree v. 1.4.2 (Rambaut 2009).

### Mating-type detection and screening for single ascospore culture

Considering that the sequence lengths of *MAT1–1-1* and *MAT1–2-1* are 708 bp and 869–880 bp, respectively, mating-type gene detection of the 380 single ascospores could be performed by observing amplicon length over an ultraviolet transilluminator after electrophoresis for first screening. This method greatly reduced the sequencing cost and accelerated mating-type detection.

### Ratio assessment of mating types and karyological analysis

The ratios of both mating types were assessed for intra- and interspecies single-spore isolates. The mating-type ratios within and among species were compared. Ascospores were stained with DAPI (4′-6-diamidino-2-phenilindole) to visualize nuclei. Pools of purified ascospores were incubated for 15 min in the staining reagent (4 mM DAPI, 100 mM Tris-HCl pH 7.5, and 20% glycerol) directly on the microscope slide. Ascospore nuclei were observed under fluorescent microscopy and laser scanning microscopy with an Olympus Fluoview FV1000 laser scanning microscope.

## RESULTS

### Identification of *MAT1–1-1* and *MAT1–2-1* genes in the genome of yellow morels

Through the BLAST analysis of the alpha-box- and HMG-containing sequences of *M. importuna* (KY508074 and KY508167) and *M. sextelata* (KY508145 and KY508228) belonging to the Elata Clade of *Morchella*, we revealed one *MAT1–1-1* and one *MAT1–2-1* genes in *Morchella* sp. *Mes-*21 genome. This was confirmed by BLAST analysis in Genbank and was best matching with *M. importuna* (APF29253 and APF29258), *Tuber indicum* (AHE80940, AHE80941) and *Penicillium kewense* (CBY44653), respectively. The primer pairs EMAT11L/EMAT11R and EMAT12L/EMAT12R were designed on the most conserved regions of the alpha-box domains of *MAT1–1-1* and HMG-box of *MAT1–2-1* genes, and then were used to amplify orthologs of the *MAT1–1-1* and *MAT1–2-1* genes from the eighty-three samples of twenty-two species.

### Genetic diversity of *MAT* idiomorphs

The most conserved regions of *MAT1–1-1* and *MAT1–2-1* were successfully amplified from the twenty-two species. *MAT1–1-1* was 708 bp and contained two exons and one intron while *MAT1–2-1* varied between 869 and 880 bp and included three exons and four introns. As the basal taxa of the Esculenta Clade, the length of *MAT1–1-1* of *M. steppicola* was similar to the ones of other species in Esculenta Clade without deletion or insertion; however, the length of *MAT1–2-1* for this species was much longer than the other species since it contained an insertion of fourteen bases. The nucleotide sequences alignment of *MAT1–1-1* included a total of 654 sites after trimming and contained 53 parsimony-informative sites and 67 singleton variable sites. Its estimated nucleotide diversity (pi) was 0.0177 with 120/654 (18.35%) variable nucleotide sites. The *MAT1–2-1* alignment consisted of a total of 827 sites after trimming and included 74 parsimony-informative sites and 84 singleton variable sites. It had an estimated nucleotide diversity (pi) of 0.0237 with 158/827 (19.10%) variable nucleotide sites.

For both *MAT* loci, higher levels of nucleotide diversity were found for interspecies than intraspecies. An lower diversity was observed in *MAT1–1-1* than *MAT1–2-1* for interspecies, but not for intraspecies (Table 1). The nucleotide diversity of *MAT1–1-1* was higher than *MAT1–2-1* in *M. clivicola*, *M. esculenta*, *M. galilaea*, *M. dunensis*, *M. yangii*, *Mes-*9, *Mes-*10, *Mes-*19, and *Mes-*20, but lower than *MAT1–2-1* in *M. amerciana*, *Mes-*6, *Mes-*15, *Mes-*21, *Mes-*22, and *Mes-*25. *Morchella palazonii* had the highest intraspecies nucleotide diversity for *MAT1–1-1* while *Mes-*21 had the highest intraspecies nucleotide diversity for *MAT1–2-1*. The protein length of *MAT1–1-1* among these species was conserved at 198 amino acids and the protein length of *MAT1–2-1* was conserved at 244 amino acids, except for *M. steppicola* with 241 amino acids.

### Mating-type ratio of single spores and reproductive modes of the species

From one to nine samples for each species were used to investigate the presence of *MAT1–1-1* and *MAT1–2-1* genes. Our results showed that both *MAT* amplicons were present in each sample of these species. To further determine whether these species were secondary homothallic or heterothallic, twenty single ascospores were isolated from each species, except for *M. steppicola*, *Mes-*24 and *Mes-*27, because no ascospores could be obtained from the culture of *M. steppicola*, the sample of *Mes-*24 was old and the samples of *Mes-*27 were immature. Each single spore always showed only a single mating type, either the *MAT1–1-1* or the *MAT1–2-1* gene. No spores harboring both mating types were found. The ratio of *MAT1–1-1*:*MAT1–2-1* of single ascospores in each species was nearby 1:1 and consistent with the null hypothesis (no deviation from a theoretical segregation ratio of 1:1, *MAT1–1-1*:*MAT1–2-1*). Therefore, the two mating types occurred equally among ascospores of a single ascomata and the reproductive modes of nineteen species were assumed to be heterothallic.

**Fig. 1:**
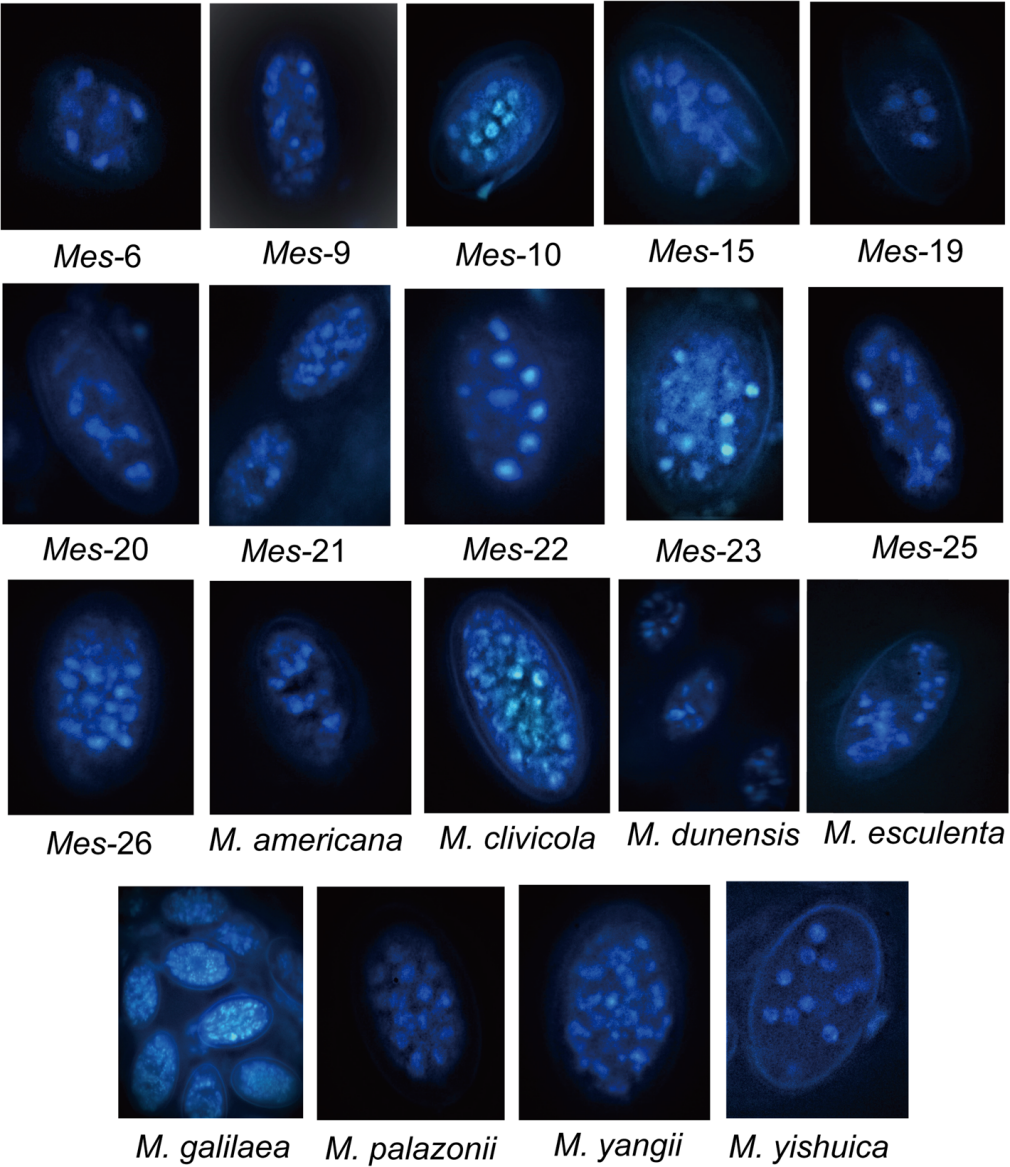
Ascospore nuclei from nineteen species stained by a fluorescent dye, 4′,6-diamidino-2-phenylindole (DAPI), and observed under the fluorescence microscope

The results of fluorescence microscopy suggest that ascospores of these species were multinuclear. The number of nuclei in each ascospores roughly divided these species into three groups (Fig. 1 and Table 3): (1) having two, four, six, eight, or more than ten nuclei, including *Mes-*6, *Mes-*9, *Mes-*19, *Mes-*20, *Mes-*22, *Mes-*23, *M. esculenta, M. dunenesis*, and *M. yangii*; (2) having more than ten nuclei, including *Mes-*10, *Mes-*15, *Mes-*21, *Mes-*25, *Mes-*26, *M. americana*, *M. palazonnii, M. yishuica,* and *M. clivicola*; and (3) having more than twenty nuclei, including *M. galilaea*.

**Table 3:** Approximate numbers of nuclei in ascospores of each species

Numbers of nucleus in ascospores	*N* = 2/4 /6 /8 /> 10	*N* > 10	*N* > 20
Species	*Mes*-6, *Mes*-9, *Mes*-19, *Mes*-20, *Mes*-22, *Mes*-23, *M. dunensis* *M. esculenta*, *M. yangii*	*Mes*-10, *Mes*-15, *Mes*-21, *Mes*-25, *Mes*-26, *M. amerciana*, *M. clivicola,* *M. palazonii,* *M. yishuica*	*M. galilaea*

**Fig. 2:**
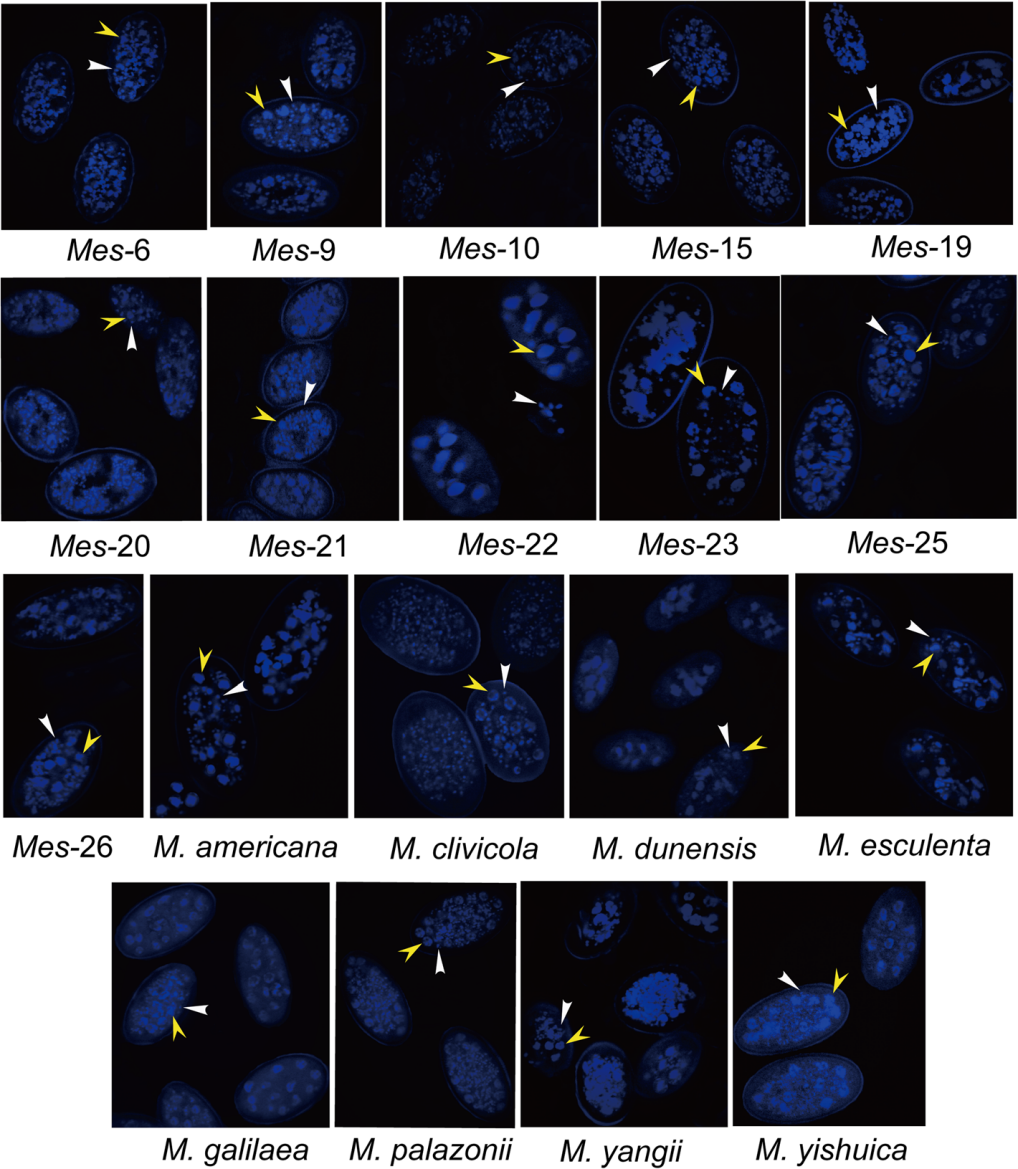
Ascospore nuclei (indicated by yellow arrowheads) and mitochondrial nucleoids (indicated by white arrowheads) from nineteen species stained by a fluorescent dye, 4′,6-diamidino-2-phenylindole (DAPI), and observed under the laser scanning confocal microscope

Notably, when observed under the laser scanning confocal microscopy, not only multi-nuclei, but also many mitochondrial nucleoids were observed in ascospores of each species (Fig. 2). Due to the many mitochondria nucleoids in ascospores, we could not further count their nuclei under the laser scanning confocal microscopy. The results from both the fluorescence microscopy and the laser scanning confocal microscopy suggest that ascospores in these species were haploid homokaryotic multinuclear that harbored many mitochondria nucleoids.

### Genealogies from reproductive gene (*MAT1–1-1* and *MAT1–2-1*) datasets and species phylogeny

**Fig. 3:**
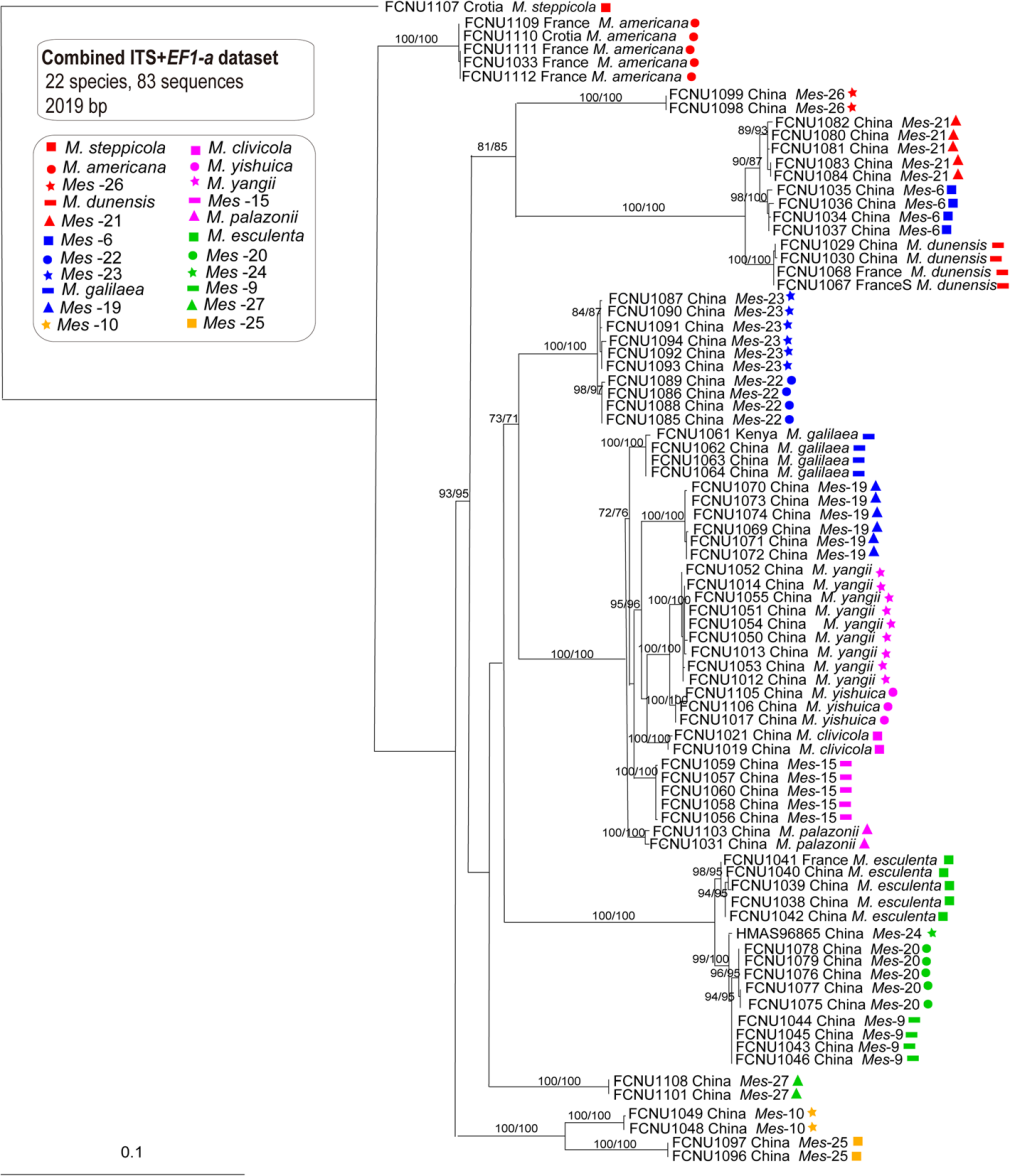
The species phylogenetic tree of the twenty-two species was inferred from 83 combined ITS+*EF1a* sequences by using RaxML and MP. Bootstrap values over 70% are shown at the nodes of the tree

**Fig. 4:**
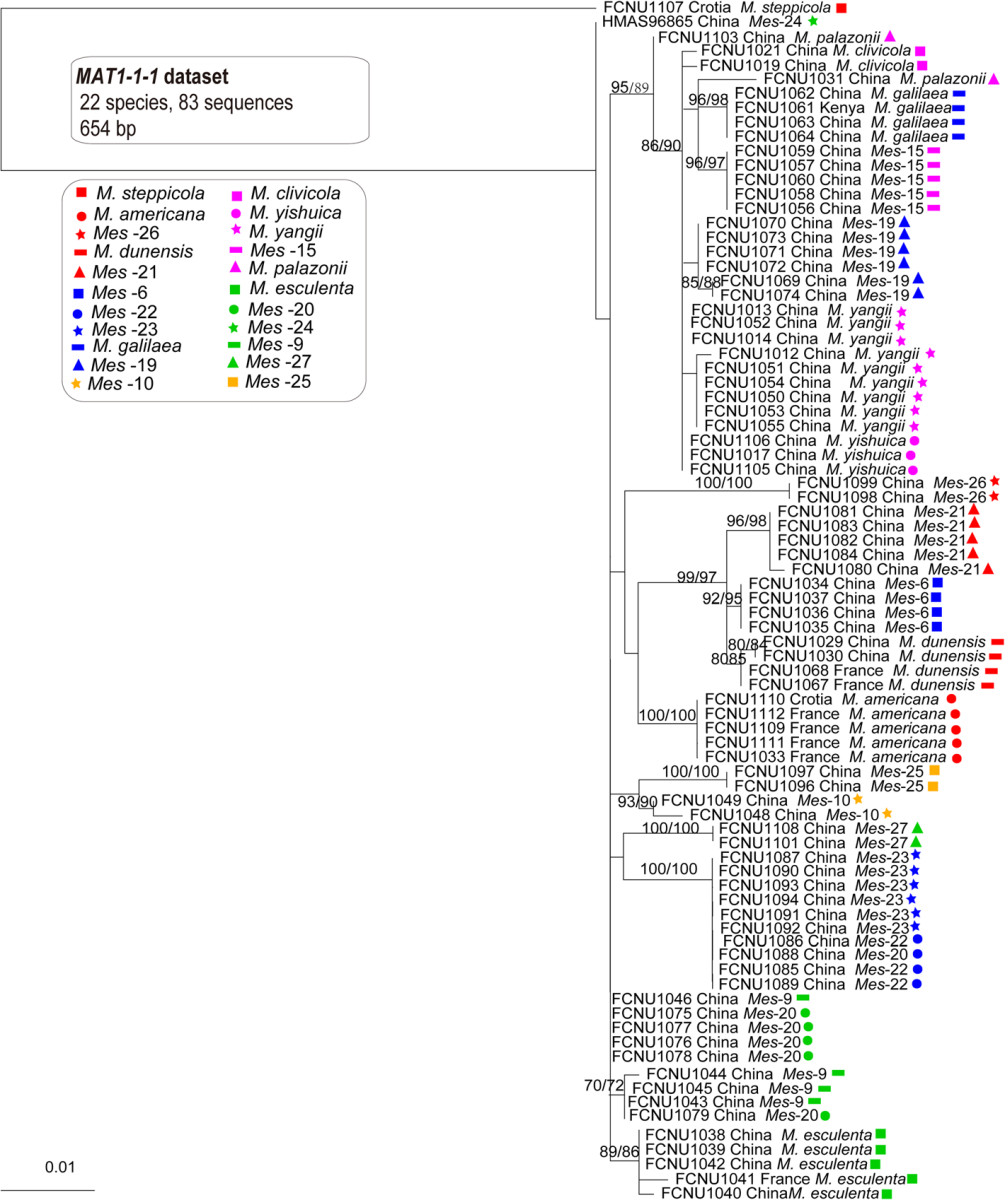
The phylogenetic tree was inferred from RaxML and MP analysis based on 83 *MAT1–1-1* sequences. Bootstrap values over 70% are shown at the nodes of the tree

**Fig. 5:**
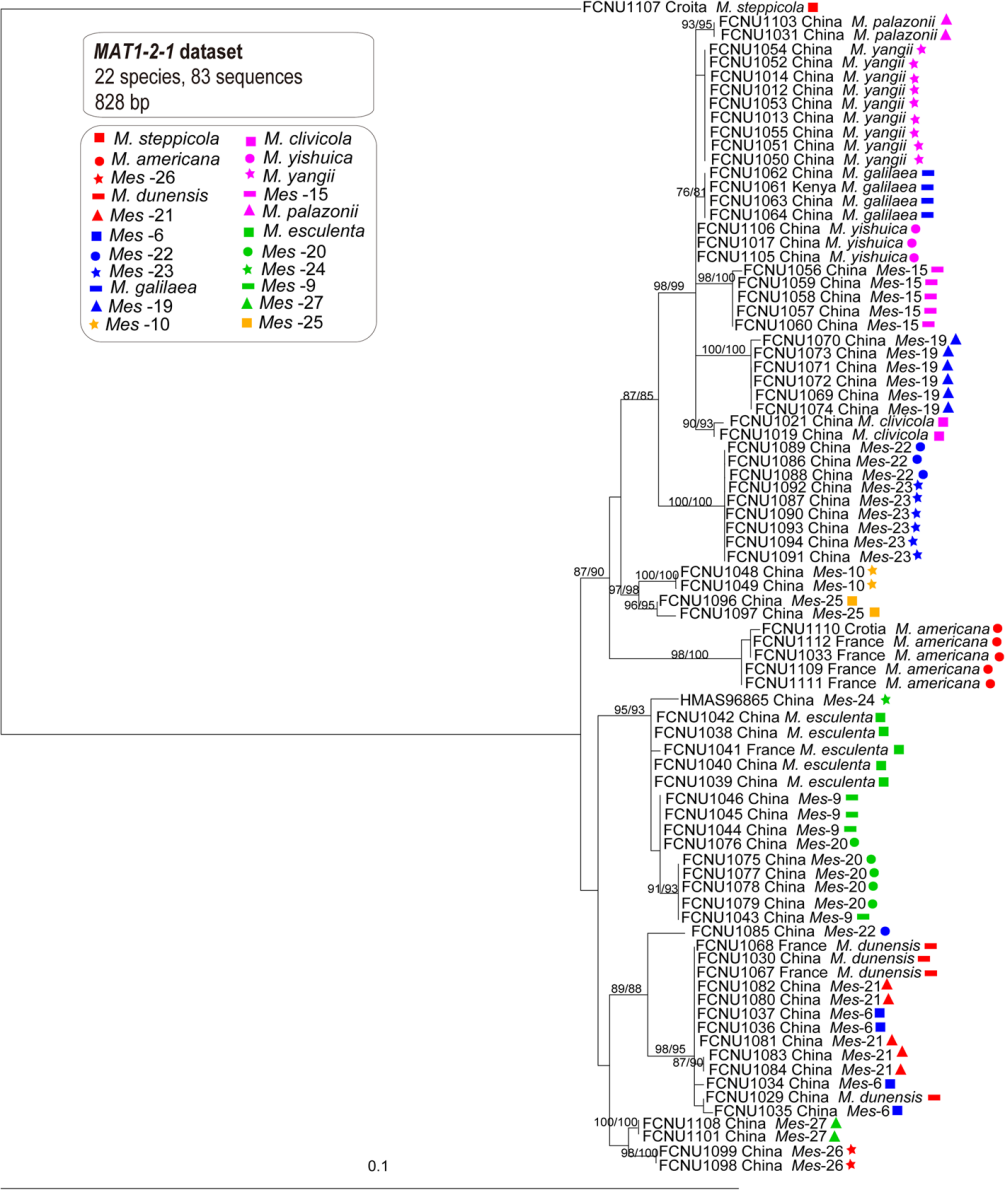
The phylogenetic relationships of the twenty-two species were inferred from 83 *MAT1–2-1* sequences by using RaxML and MP. Bootstrap values over 70% are shown at the nodes of the tree

A species phylogeny framework for the twenty-two species (Fig. 3) was constructed using the ITS and *EF1-a* combined dataset with 2019 bp using the maximum likelihood and parsimony phylogenetic method which was also used for inferring the genealogies of both *MAT1–1-1* and *MAT1–2-1* datasets, resepctively with 654 bp and 828 bp (Figs. 4 and 5). The phylogenetic trees of *MAT1–1-1* and *MAT1–2-1* were used to represent the reproductive genes phylogeny of these species (Figs. 4 and 5). Both the MP and ML trees of *MAT1–1-1* and *MAT1–2-1* had the similar topological phylogenies, but their backbones were weakly supported. The species trees supported that each of the twenty-two species formed a monophyletic group; however, the phylogenetic trees for *MAT1–1-1* and *MAT1–2-1* displayed several topological conflicts with the species tree and between each other (Figs. 3, 4 and 5).

First, the topological positions of the species differed between the *MAT1–1-1* genealogy and the species tree (Figs. 3 and 4). In the species tree, *M. clivicola*, *M. yishuica*, *M. yangii* and *Mes-*20, respectively formed well-supported monophyletic groups, in contrast to the *MAT1–1-1* genealogy, in which samples from these species did not form monophyletic groups. Samples from *Mes-*22 and *Mes-*23 could be well divided into two monophyletic groups with high support (ML and MP bootstrap > 80%) in the species tree where the relationship between them was indicated to be very close; however, the *MAT1–1-1* genealogy supported samples from *Mes-*22 and *Mes-*23 belonging to one species with 100% ML and MP bootstrap value.

Second, the topological positions of the species differed between the *MAT1–2-1* genealogy and the species tree. In the species tree, *M. dunensis*, *Mes-*6 and *Mes-*21 respectively formed well-supported monophyletic groups (ML and MP bootstrap > 85%) and were closely related sister groups; however, in the *MAT1–2-1* genealogy, all samples from these three species formed a well-supported monophyletic group (ML and MP bootstrap = 98%). In the species tree, *Mes-*9 and *Mes-*20 could each form a well-supported monophyletic group, in contrast to the *MAT1–2-1* genealogy, in which samples from two species did not form monophyletic groups. Samples from *Mes-*22 and *Mes-*23 could be well divided into two monophyletic groups with high support (ML and MP bootstrap > 80%) in the species tree; however, the *MAT1–2-1* genealogy clustered most of the samples from both *Mes-*22 and *Mes-*23 into one monophyletic group (ML and MP bootstrap = 100%), except for FCNU1085, which was grouped together with *M. dunensis*, *Mes*-6 and *Mes*-21 and presumed to be a hybrid individual.

Third, the placement of these species also differed between the *MAT1–1-1* and *MAT1–2-1* genealogy (Figs. 4 and 5). Samples from *Mes-*9 and *Mes-*20 did not form monophyletic groups in either the *MAT1–1-1* genealogy or the *MAT1–2-1* genealogy, both of which is in conflict with the species phylogeny, while they showed different topological positions between each other as well. Samples from *Mes-*10 had no variation on *MAT1–2-1* sequences while they highly varied on *MAT1–1-1* sequences. The same phenomenon was found in *M. palazonii* and *M. dunensis*. Samples from *Mes-*25 had no variation on *MAT1–1-1* sequences while they highly varied on *MAT1–2-1* sequences. The same phenomenon was found in *M. americana* and *Mes-*6.

### Genealogies from non-reproductive (*F1* and IGS) datasets and species phylogeny

**Fig. 6:**
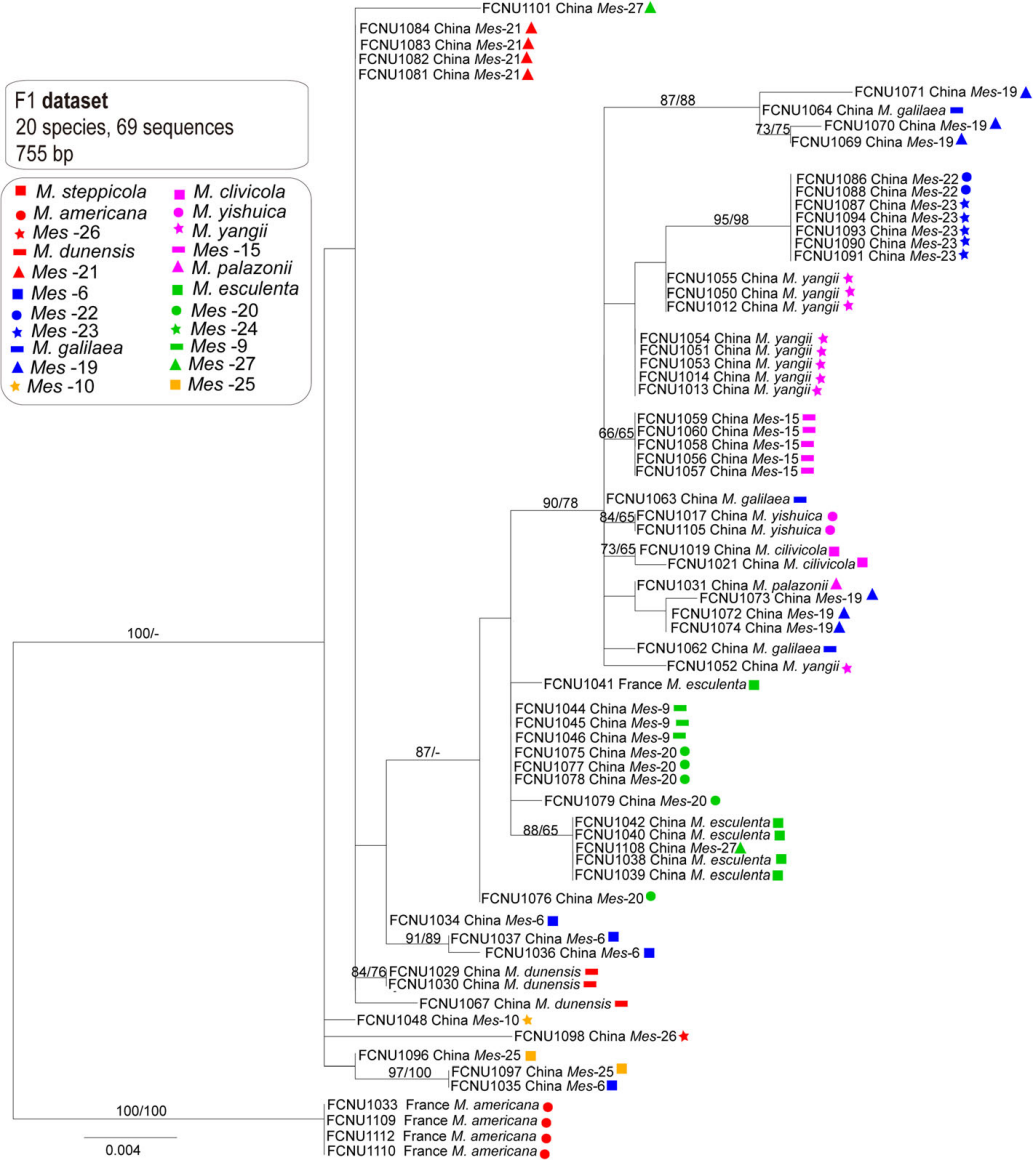
The phylogenetic relationships were inferred from 69 IGS sequences based on RaxML and MP. Bootstrap values over 70% are shown at the nodes of the tree

**Fig. 7:**
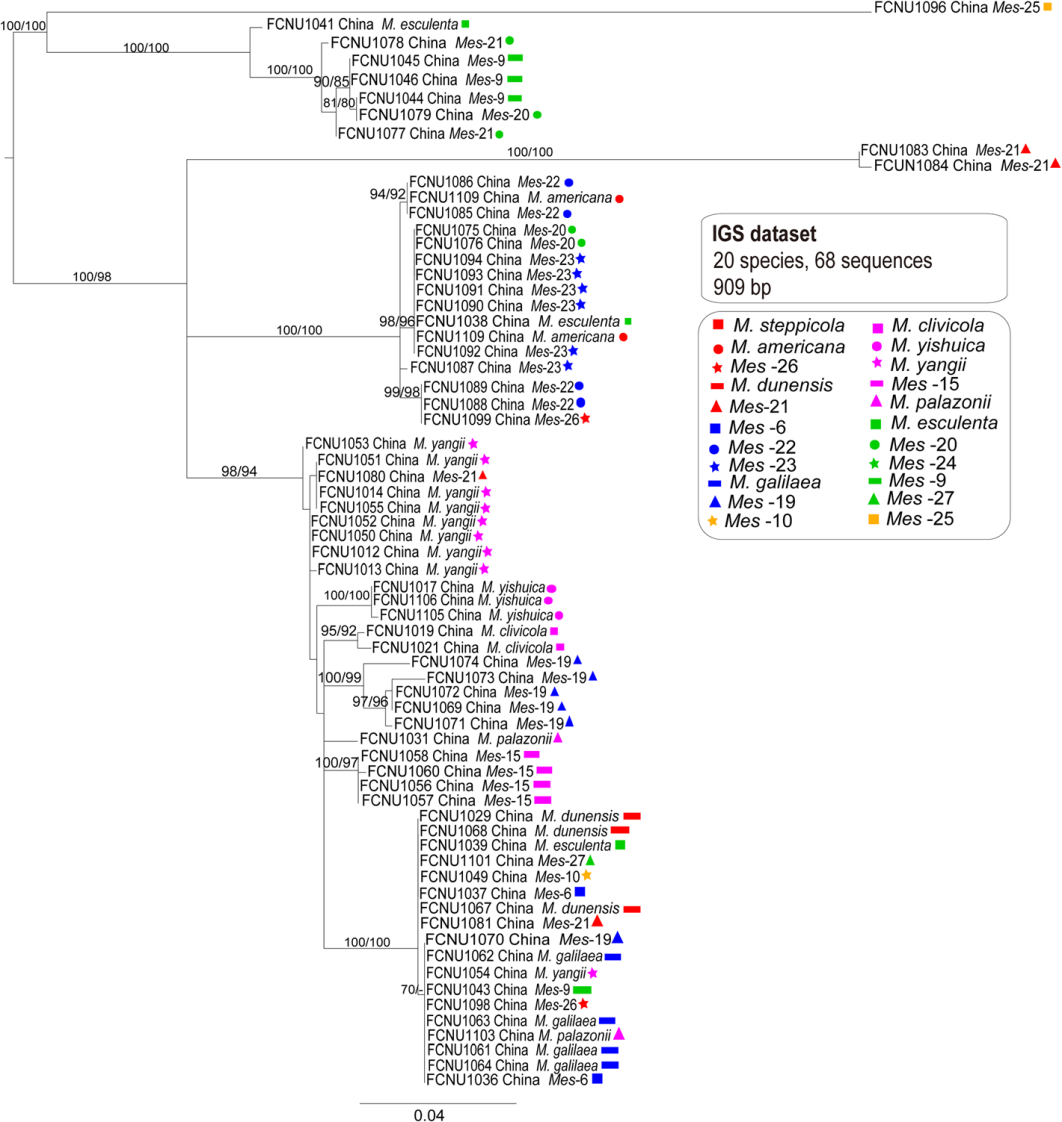
The phylogenetic tree was inferred from 68 *F1* sequences by using RaxML and MP. Bootstrap values over 70% are shown at the nodes of the tree

The primers of *F1* and IGS developed in this study did not result in an amplification product for fourteen and fifteen of the samples used in the present study, respectively, although we redesigned new primers and extracted DNA several times to amplify these samples, we always failed. The final aligned lengths of *F1* and IGS datasets used for generating phylogenetic trees were, respectively, 755 bp and 909 bp. The MP and ML trees of *F1* and IGS had different topological structures. Though the same samples were always clustered together in both MP and ML trees of *F1*, the placements of branches always changed and their backbones were weakly supported. For the MP and ML trees of IGS, it’s the similar case as the ones of *F1*. Conflicts were found among the genealogies of *F1* (Fig. 6) and IGS (Fig. 7) and the species phylogeny (Fig. 3).

In the *F1* genealogy, samples from *M. steppicola* and *Mes-*24 failed to generate amplicons, and only one sample from each of the *Mes-*10, *Mes-*26, and *M. palazonii* obtained *F1* amplicons, so when conflicts between the *F1* phylogeny and the species phylogeny were considered, these five species were excluded. Samples from *M. americana*, *M. clivicola*, *M. yishuica*, *Mes-*15, and *Mes-*27 each formed a monophyletic group in the *F1* genealogy as well as in the species phylogeny, although the placements of the five species differed between the *F1* genealogy (Fig. 6) and the species tree (Fig. 3). Samples of *Mes-*6 did not form a monophyletic group in the *F1* genealogy and the sample FCNU1035 from this species formed a monophyletic group together with FCNU1097 of *Mes-*25 with high support (ML and MP bootstrap > 95%). This indicated that some hybridization events might have happened between *Mes-*6 and *Mes-*25, and that FCNU1035 might be a hybrid. Samples from *M. esculenta*, *Mes-*9 and *Mes-*20 did not form monophyletic groups in the *F1* genealogy. Samples of *M. galilaea* also did not form a monophyletic group as well as in the *F1* genealogy, whereas one sample of this species, FCNU1064, from this species formed a monophyletic group with three samples of *Mes-*19, namely FCNU1069, FCNU1070 and FCNU1071. This indicated that some hybridization events might have happened between *M. galilaea* and *Mes-*19, and that FCNU1064 might be a hybrid. Samples from *M. dunensis*, *M. yangii* and *Mes-*21 did not form monophyletic groups, and samples from *Mes-*22 and *Mes-*23 formed a monophyletic group in the *F1* genealogy.

Samples from *M. steppicola* and *Mes-*24 failed to generate IGS amplicons and only one of the samples of *M. americana*, *Mes-*10, *Mes-*25, and *Mes-*27 had amplicons from IGS, thus, these five species were excluded. Samples from *M. clivicola*, *M. yishuica* and *Mes-*15 each formed a monophyletic group in the IGS genealogy as well as in the species phylogeny. Samples from *M. esculenta*, *M. palazonnii*, *M. dunensis*, *M. yangii*, *Mes-*6, *Mes-*9, *Mes-*19, *Mes-*20, *Mes-*21, *Mes-*22, *Mes-*23 and *Mes-*26 did not form monophyletic groups in the IGS genealogy. One sample from *M. dunensis*, one sample from *M. esculenta*, two samples from *Mes-*20 and five samples from *Mes-*23 formed a monophyletic group with high support (ML and MP bootstrap > 95%). Three different IGS sequences were obtained from the four samples of *Mes-*21 (FCNU1080, FCNU1081, FCNU1083, and FCNU1084), and the IGS sequence of FCNU1080 and those of the three samples of *M. yangii* were clustered into a monophyletic group together. Three different IGS sequences from three samples of *M. esculenta* (FCNU1038, FCNU1039 and FCNU1041) and two from two samples of *Mes*-26 (FCNU1098 and FCNU1099) were obtained. The IGS sequences of FCNU1088 and FCNU1089 of *Mes*-22 and that of FCNU1099 of *Mes*-26 were grouped into a monophyletic group with high support (ML and MP bootstrap > 95%), suggesting that some hybridization events might have happened between *Mes*-22 and *Mes*-26.

### Genealogical conflicts among *MAT1–1-1*, *MAT1–2-1*, *F1*, IGS, and species phylogeny

Conflicts were identified among the *MAT1–1-1*, *MAT1–2-1*, *F1*, IGS, and species phylogenies. The percentages of species in *MAT1–1-1*, *MAT1–2-1*, *F1,* and IGS genealogies which were in conflicted with species phylogeny were 30, 35, 66.7 and 81.3%, respectively (Table 4). The IGS genealogy had the most conflicts with species phylogeny, followed by *F1*, *MAT1–2-1*, and *MAT1–1-1*. Herein, the conflicts mainly referred to samples forming a monophyletic group in the species phylogeny but not in the four other genealogies. It is interesting that *M. yishuica* and *Mes-*15 did not have conflicts among the four genes genealogies and with the species phylogeny. For *M. americana* and *Mes-*27, except IGS with only one sample having amplicons in both species, no conflicts were found in the *MAT1–1-1*, *MAT1–2-1* and *F1* genealogies. Among the four genes, the success rate of amplification in IGS was the lowest.

**Table 4:** Phylogenetic conflicts present in each species according to these gene genealogies

Species	Species phylogeny	*MAT1–1-1*	*MAT1–2-1*	*F1*	IGS
*M. amerciana*	×^a^	×	×	×	O^c^
*M. clivicola*	×	√^b^	×	×	×
*M. dunensis*	×	×	√	√	√
*M. esculenta*	×	×	×	√	√
*M. galilaea*	×	×	×	√	√
*M. palazonii*	×	×	×	O	√
*M. steppicola*	O	O	O	N^d^	N
*M. yangii*	×	√	×	√	√
*M. yishuica*	×	×	×	×	×
*Mes* − 6	×	×	√	√	√
*Mes* − 9	×	√	√	√	√
*Mes* − 10	×	×	×	O	O
*Mes* − 15	×	×	×	×	×
*Mes* − 19	×	×	×	√	√
*Mes* − 20	×	√	√	√	√
*Mes* − 21	×	×	√	√	√
*Mes* − 22	×	√	√	×	√
*Mes* − 23	×	√	√	√	√
*Mes* − 24	O	O	O	N	N
*Mes* − 25	×	×	×	√	O
*Mes* − 26	×	×	×	O	√
*Mes* − 27	×	×	×	×	O
Total conflicts	/	30%	35%	66.7%	81.3%

^a^: No conflicts were detected in this species according to the current gene genealogy

^b^: Conflicts were found in this species according to this gene genealogy

^c^: Only one sample of this species obtained amplicons from this gene

^d^: No sample of this species obtained amplicons from this gene

The phylogenies of reproductive genes (*MAT1–1-1* and *MAT1–2-1*) showed more resolution in these yellow morel species than the non-reproductive genes (*F1* and IGS) (Figs. 4, 5, 6 and 7). In the *MAT1–1-1* and *MAT1–2-1* genealogies, fourteen and fifteen well-supported branches consistent with species phylogeny were formed, respectively (Figs. 4 and 5). In the *F1* and IGS genealogies, only five or three well-supported branches and consistent with the species phylogeny were formed, respectively (Figs. 4 and 5).

## TAXONOMY

Not applicable.

## DISCUSSION

The availability of the genome sequence of *Mes-*21 created the opportunity to unveil *MAT* loci and mating strategies of the Esculenta Clade in the genus *Morchella*. In this study, primers were developed to amplify and sequence *MAT1–1-1* and *MAT1–2-1* genes from twenty-two species of the Esculenta Clade. Phylogenies resulting from these *MAT* sequences were compared with those resulting from the analyses of *F1* (Du et al. 2016), IGS and a species phylogeny framework of *Morchella* and revealed multiple conflicts among them.

### Comparison of *MAT* loci provides insights into the evolution of the genus *Morchella*

In the Elata Clade, the length of *MAT1–1-1* (729–736 bp) is longer than *MAT1–2-1* (398–408 bp) (Du et al. 2017). In the Esculenta Clade, the current results indicate that *MAT1–1-1* (651–654 bp) is shorter than *MAT1–2-1* (813–827 bp). This difference indicated that the mating-type genes in the Elata and Esculenta Clades appear to have a differential evolutionary history. Analyses of the *MAT* sequence showed higher levels of nucleotide diversity (pi) in both *MAT1–1-1* (0.0178) and *MAT1–2-1* (0.0236) within these species than intraspecies nucleotide diversity (pi). This indicated that *MAT* genes evolved rapidly among morel species. Strong purifying selection against deleterious mutations were suggested in *MAT* genes (Turgeon 1998; Heitman 2015), which probably resulted in the low intraspecific polymorphism observed in *MAT* genes of morels. Additionally, *MAT1–2-1* was found to be more variable than *MAT1–1-1* among the twenty-two species, which is consistent with the results in the Elata Clade (Du et al. 2017). Du et al. (2017) suggested that different sexual competences of *MAT1–1-1* and *MAT1–2-1* might exist in black morels which deserved to be investigated in yellow morels.

### Dominant heterothallic reproductive modes in the genus *Morchella*

The *MAT1–1-1* and *MAT1–2-1* genes are scarcely conserved through different classes of fungi which is the main obstacle to identify sexual genes and decipher their reproductive modes in morels. Recently, the sequence of the *M. eximia* genome allowed the first set of MAT genes, namely *MAT1–1-1* and *MAT1–2-1*, to be mined from fourteen black morel species in the genus *Morchella* and proved that the reproductive modes of these species are mainly heterothallic. However, at that time, *MAT1–1-1* and *MAT1–2-1 g*enes were not obtained from yellow morel species after multiple attempts due to the conservation of *MAT* genes (Du et al. 2017). To this end, based on the genome of *Mes-*21, we employed PCR primers designed for the most conserved domains, namely the alpha and HMG domains, of *Mes-*21 *MAT* genes, to amplify the *MAT* genes from twenty-two yellow morel species. Although no single ascospores were obtained from *M. steppicola*, *Mes*-24, and *Mes*-27, these three species are suggested to be heterothallic based on the fact that all species in the genus *Morchella* investigated thus far are heterothallic and that both MAT genes were obtained from their strains.

Heterothallic reproductive modes of the twenty-two species of the Esculenta Clade were supported by the evidence that amplicons specific to both *MAT* genes were evenly obtained by PCR from he single spores collected. Combined with the reported heterothallic reproductive modes of fourteen black morel species in the Elata Clade (Du et al. 2017), species in the genus *Morchella* might mainly be heterothallic. The prevalence of heterothallism as mating strategy in fungi is an important feature with implications on the genetic variability and evolutionary potential of a species (Lopes et al. 2018).

Similar to the results reported in black morel species (Du et al. 2017), ascospores of yellow morel species were shown to be haploid homokaryotic multinuclear by DAPI-staining methods under both the fluorescence microscopy and laser scanning confocal microscopy. According to the number of nuclei in the ascospores, these species could be divided into three kinds. *Morchella galilaea*, the widest distributed species of the Esculenta Clade had the most nuclei in ascospores among these species. It is possible that the number of ascospore nuclei might be positively correlated with the distribution range of the species, as the number of nuclei in ascospores might contribute to their vitality and spreading ability resulting in their wide distribution. However, more samples of *M. galilaea* and other morel species should be collected for further study and comparison.

In addition to nuclei, many mitochondria nucleoids were observed in ascospores of these yellow morel species by laser scanning confocal microscopy, similar to what was observed in other fungi by DAPI-staining (Oakley and Rinehart 1985; Chen and Butow 2005; Kucej and Butow 2007). The presence of many mitochondria nucleoids in black morels ascospores was previously shown. In fungi, mitochondrial–nuclear interactions are involved in the control of aging processes and the age-related changes in the mitochondrial DNA are proven to be part of the process leading to organismal degenerations (Osiewacz and Kimpel 1999; Osiewacz 2002). Whether the number of mitochondria nucleoids in morel ascospores would be related to morel strain degradation is not known yet and should be studied in the future.

### Conflicts among the genealogies of four targeted genes and species phylogeny

In this study, we assessed the capability of gene trees from reproductive genes (*MAT1–1-1* and *MAT1–2-1*) and non-reproductive genes (*F1* and IGS) to resolve species relationships by comparing genes trees with a species phylogeny framework of the twenty-two species inferred from ITS and *EF1-a* combined datasets which were derived from multi-locus analyses and were previously used in studies of the genus *Morchella* (Taşkın et al. 2010; O’Donnell et al., 2011; Du et al. 2012, 2019b; Loizides et al. 2016; Baroni et al. 2018). One of the findings in this study is the topologies for the reproductive gene trees (mating-type) and non-reproductive genes (*F1* and IGS) were in conflict with the species phylogeny framework of the Esculenta Clade. Conflicts between the gene tree genealogies and the species phylogeny could be signatures of evolutionary processes such as hybridization, introgression, gene transfer and incomplete lineage sorting (Degnan and Rosenberg 2009).

1. Conflicts between reproductive genes and species trees

Individual *MAT1–1-1* and *MAT1–2-1* gene genealogies resolved most of the species proving their potential usefulness as phylogenetic markers for species delimitation in the Esculenta Clade (Figs. 4 and 5). Although *MAT1–2-1* has previously been considered to be a better phylogenetic marker than *MAT1–1-1* (Martin et al. 2010), here we found both MAT genes to be equally efficient in resolving species in the Esculenta Clade, with six species conflicting with the species phylogeny in the *MAT1–1-1* genealogy and seven species in the *MAT1–2-1* genealogy.

Taylor et al. (2000) proposed that conflicts among gene trees are possibly the result of recombination among individuals and that the transition from concordance to conflict determines the limits of the species. This is the principle of phylogenetic species recognition widely which is applied to fungi and specifically to *Morchella*. O’Donnell (2000) suggested that gene genealogy concordance is well suitable to identify hybrid individuals because hybrids should be grouped with different species in different single-gene genealogies. Several studies revealed that phylogenies of mating-type genes are in general consistent with those of other genes, such as ITS, *EF1-a*, *Beta-tubulin* and *RPB2* (Turgeon 1998; Waalwijk et al. 2002; O’Donnell et al. 2004; Inderbitzin et al. 2005; Yokoyama et al. 2006; Duong et al. 2013; Du et al. 2017; Lopes et al. 2017, 2018), while other studies found they are conflicting (Wik et al. 2008; Strandberg et al. 2010).

Conflicts among genealogies of *MAT1–1-1* and *MAT1–2-1* and the species phylogeny indicated that some recombination or hybridization events probably occurred between *Mes-*20 and *Mes-*9. As sister species with a similar distribution area, *Mes-*20 and *Mes-*9 have the geographical advantage and potential incomplete reproductive isolation for hybridization to occur. The fact that both *MAT1–1-1* and *MAT1–2-1* genealogies showed the same conflicts with the species tree for *Mes-*20 and *Mes-*9 is an important finding, indicating that the two mating types likely introgressed from a single ancestral source rather than independently from different ancestors. This pattern suggested a non-random process of acquisition of the MAT alleles in the evolutionary history of the two species.

*MAT* genes are divergent and known to evolve quickly (Turgeon 1998). However, the *MAT1–1-1* and *MAT1–2-1* sequences of *Mes-*22 and *Mes-*23 samples had no differences between the two lineages, suggesting that *Mes-*22 and *Mes-*23 retained the ancestral character of interbreeding due to recent divergence. Thus, in this case, by having identical *MAT* gene sequences as a proxy for mating, they would in fact represent distinct phylogenetic species. Additionally, there was the possibility that incomplete lineage sorting caused the current patterns of *Mes-*22 and *Mes-*23 in the *MAT1–1-1* and *MAT1–2-1* genealogies. In the genealogy of *MAT1–2-1*, *Mes-*6, *Mes-*21 and *M. dunensis* formed a monophyletic group with almost identical *MAT1–2-1* gene sequences. Having identical *MAT* gene sequences as a proxy for mating was suggested to be the ancestral character of interbreeding. *Morchella clivicola*, *M. palazonii,* and *M. yangii* did not form monophyletic groups in the genealogy of *MAT1–1-1*. In addition, either of the *MAT* genes genealogies, such as *MAT1–2-1* for *Mes-*6, *Mes-*21, and *M. dunensis*, and *MAT1–1-1* for *M. clivicola*, *M. palazonii,* and *M. yangii*, had conflicts with the species tree indicating that the two mating types independently evolved and one introgressed from different ancestors. This pattern suggested a random process of acquisition of the *MAT* alleles in the evolutionary history of these six species.

Unlike the phylogenies of heterothallic *MAT* loci shown in the Elata Clade (Du et al. 2016), the phylogeny of *MAT* loci in the Esculenta Clade presented here contained a few well-supported deviations from the species tree. This could be taken as evidence that lateral gene-flow existed in Esculenta Clade. Furthermore, the existence of conflicts between the phylogenies of *MAT1–1-1* and *MAT1–2-1* provided insights into the evolution of the Esculenta Clade and indicated that both loci evolved independently.

2. Conflicts between non-reproductive genes (*F1* and IGS) and the species phylogeny reveal potential hybridization

The conflicts between the genealogies of MAT genes and species phylogenies called for a further analysis of species relationship and the evolution of mating-type loci in *Morchella*. One way to improve the resolution of the species tree is to increase the number of loci used in the study. The *F1* fragment was firstly used in the Elata Clade of the genus *Morchella* for population studies and some potential hybridization or gene transfer events were revealed in several species (Du et al. 2016). The phylogeny of intergenic spacer region (IGS) was reported to conflict with the phylogeny of *EF1-a* in the genus *Fusarium* (Mbofung et al. 2006; Silva et al. 2014). By comparing the reproductive (*MAT1–1-1* and *MAT1–2-1*) and the non-reproductive (*F1* and IGS) datasets, we tested whether the conflicts are inherent only to the reproductive genes or they also exist in other parts of the genome in species of the Esculenta Clade. According to the above results, the phylogenies of both *F1* and IGS were shown to conflict with each other and with the species phylogeny (Figs. 6 and 7).

In *F1*, only five species were in consistent with the species phylogeny. Strong conflicts were detected between the genealogy of *F1* and the species phylogeny in the Esculenta Clade, which were more serious than those reported in the Elata Clade (Du et al. 2016). Three samples (FCNU1062, FCNU1063, and FCNU0164) from *M. galilaea* generated three kinds of *F1* sequences and the phylogenetic position of FCNU1064 inferred from the *F1* phylogeny was clustered with samples of *Mes-*19. This indicated that some hybridization events might have happened between *Mes-*19 and *M. galilaea*. Hybridization processes in nature occur between pairs of taxa within a species complex (Garbelotto and Gonthier 2013). *Morchella galilaea* and *Mes*-19 are related species and both are widely distributed in China, which gives them the chance to meet in sympatry or allopatry. It is interesting to note that interspecific mating and hybridization has been reported for fungi such as *Ophiostoma* spp., *Heterobasidion* spp., *Melampsora* spp. and *Microbotryum* spp. (Chase and Ullrich 1990; Brasier et al. 1998; Newcombe et al. 2000; Gonthier et al. 2007; Gladieux et al. 2011). Hybridization occurred through secondary contact following initial divergence in allopatry for some fungi (e.g., *Microbotryum*) (Gladieux et al. 2011). The sample FCNU1035 from *Mes-*6 had the same *F1* sequence as the sample FCNU 1097 from *Mes-*25 and they were grouped into a monophyletic group. Thus, we assume that horizontal gene transfer or hybridization events might have happened between *Mes-*6 and *Mes-*25.

In IGS, only three species were in consistent with the species phylogeny. A high degree of conflicts were shown between the genealogy of IGS and the species phylogeny. According to the species phylogeny, different kinds of IGS sequences were obtained from samples belonging to the same species. Frequent potential hybridization or horizontal gene transfer events of IGS were suggested to exist in the Esculenta Clade. When they detected conflicts between phylogenies of IGS and *EF1-a* in the genus *Fusarium,* Mbofung et al. (2006) proposed that unequal rates of evolution between loci and incomplete concerted evolution within loci could be among the factors responsible for the observed discrepancies. Most likely, these factors also contributed to the conflicts shown here.

With the increasing availability of molecular data, phylogenetic trees generated from different genes are often recognized to have conflicting branching patterns (Maddison 1997; Nichols 2001; Pamilo and Nei 1988). Davin et al. (2018) suggested that different genetic markers could yield conflicting estimates of the species phylogeny. In recent years, genome-scale sequence data have become increasingly available in phylogenetic studies for understanding the evolutionary histories of species (Liu et al. 2015). Phylogenetic analyses of genomic data revealed that different genes within a genome can have different evolutionary histories, i.e. phylogenetic conflicts (Spatafora et al. 2017; Shen et al. 2017). However, research on the sources of these conflicts, including incomplete lineage sorting, hybridization and horizontal gene transfer, as well as on the detection and characterization of these conflicts according to phylogenetic inference, is still in the early stage (Mirarab et al. 2014; Spatafora et al. 2017).

We presumed that the conflicts among the phylogenies of *F1*, IGS, and the species phylogeny were likely due to gene transfer and hybridization. Gene transfer is often suggested as an explanation for incongruencies between gene trees and species trees; however, although it is widely assumed to be more common in prokaryotes, it’s not considered to be a common phenomenon in eukaryotes (Dujon 2005; Syvanen 1994; Gogarten 2003; Galtier and Daubin 2008). Hybridization is now widely recognized to be an important evolutionary process which might play a crucial role in speciation (Gross and Rieseberg 2005; Mallet 2007; Schumer et al. 2014; Taylor and Larson 2019). Hybridization followed by reproductive isolation has been reported to contribute to rapid speciation of yeast (Leducq et al. 2016) and the same has been supposed to occur in some filamentous fungi (Kohn 2005; Gladieux et al. 2014; Sillo et al. 2019). Du et al. (2012) reported the 85.2% of the species in the Esculenta Clade (including almost 90% of the Chinese species lineages) diversified and went through rapid speciation in East Asia since the middle Miocene, which might have contributed to the potential hybridization and gene transfer events detected in the Esculenta Clade. Of note, we could not exclude the possibility that conflicts arising from gene trees and species phylogeny might be due to weak phylogenetic signal for *F1* and IGS.

## CONCLUSIONS

The results from our study showed that heterothallism is the reproductive mode in the Esculenta Clade and in the genus *Morchella*. The primers designed for *MAT* loci in the Esculenta Clade supplemented the primers previously developed for the Elata Clade (Du et al. 2017). *MAT* genes of the Elata Clade had similar topologies to the species phylogeny in the study of Du et al. (2017). However, the present study indicated that *MAT* genes of the Esculenta Clade were in conflict with species tree. Divergent evolutionary patterns were suggested for *MAT* genes between the Esculenta and the Elata Clades. Complex evolutionary trajectories of *MAT1–1-1*, *MAT1–2-1*, *F1* and IGS in the Esculenta Clade were highlighted. Our findings contribute to a better understanding of the importance of hybridization and gene transfer in *Morchella* and especially for shaping reproductive modes during its evolutionary process. The genus *Morchella* will be useful for studying the complexities and evolution of mating types and genomes in the *Ascomycota*.

## Data Availability

Sequences generated in the present study have been deposited in GenBank under accession numbers MN513618-MN513997 and MN513426-MN513494. The full alignments of these datasets were submitted to the TreeBASE (25132). The genome data of Mes-*21* generated in this project has been deposited at DDBJ/ENA/GenBankunder the accession WSNQ00000000. The version described in this paper is version WSNQ01000000.

## Abbreviations

*EF1-a*: Translation elongation factor 1-a.
*F1*: Partial sequences of histone acetyltransferase ELP3.
IGS: 28S–18S ribosomal intergenic spacer region.
ITS: Nuc-rDNA internal transcribed spacer region.
MAT: mating type.
