# Correction to: Heterothallism and potential hybridization events inferred for twenty-two yellow morel species

**DOI:** 10.1186/s43008-022-00096-0

**Published:** 2022-05-19

**Authors:** Xi-Hui Du, Dongmei Wu, Heng Kang, Hanchen Wang, Nan Xu, Tingting Li, Keliang Chen

**Affiliations:** 1 College of Life Sciences, Chongqing Normal University, Chongqing, 401331 China; 2 Biotechnology Research Institute, Xinjiang Academy Agricultural Reclamation of Sciences, Shihezi, 832000 China; 3 Institute of Applied Mycology, Huazhong Agricultural University, Wuhan, 430070 Hubei China

## Correction to: IMA Fungus (2020) 11:4 10.1186/s43008-020-0027-1

Following the publication of the original article (Du et al. 2020), we were notified of two mistaken pairs of primer sequences in Table 2, as shown below.

Incorrect sequence:
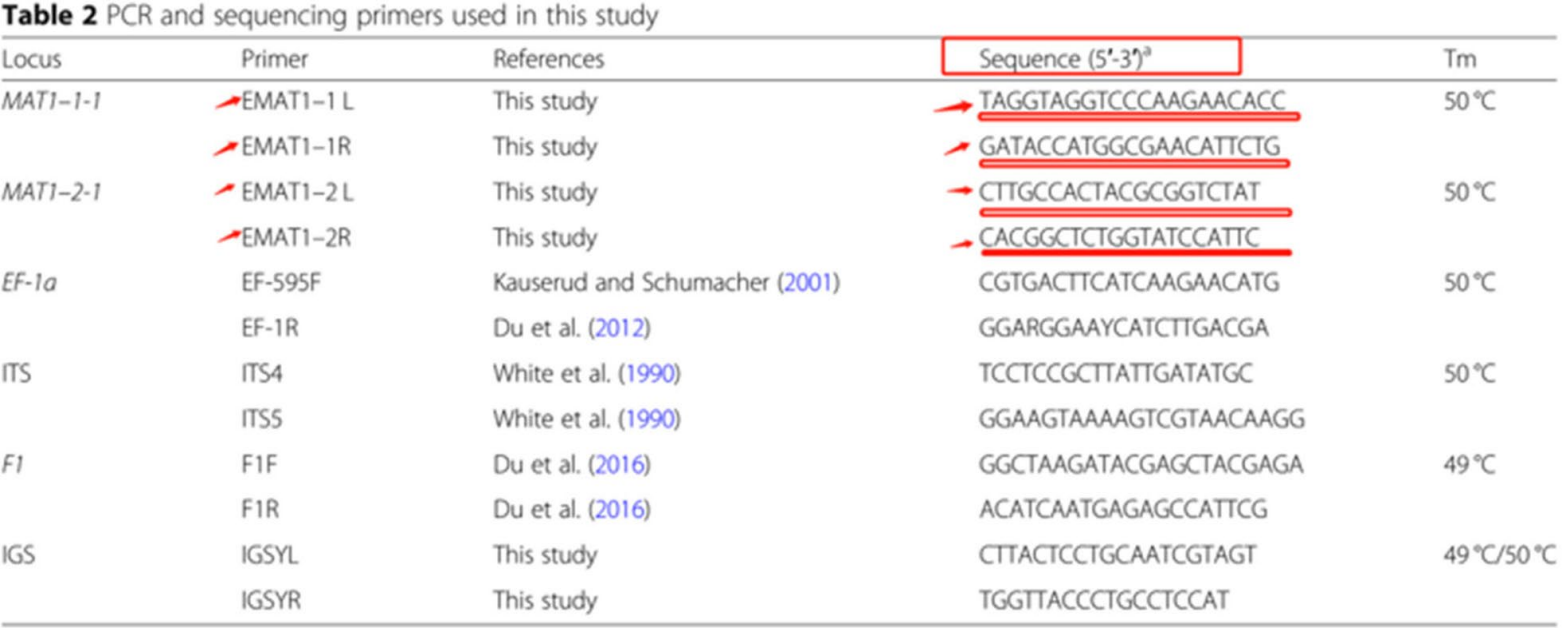


Corrected sequence:

EMAT1–1L: TGAGTCCGTTATGATTCTGG

EMAT1–1R: GGACCATTCGCTTTCTCATA

EMAT1–2L: GATATGCTCACCAACCGTAA

EMAT1–2R: TACGATCGAATAATGGCTCC

The original article has been corrected.
